# Development of National Newborn Screening Quality Indicators in the United States

**DOI:** 10.3390/ijns5030034

**Published:** 2019-09-12

**Authors:** Careema Yusuf, Marci K. Sontag, Joshua Miller, Yvonne Kellar-Guenther, Sarah McKasson, Scott Shone, Sikha Singh, Jelili Ojodu

**Affiliations:** 1 Association of Public Health Laboratories, Silver Spring, MD 20910, USA; sikha.singh@aphl.org (S.S.); jelili.ojodu@aphl.org (J.O.); 2 Center for Public Health Innovation at CI International, Littleton, CO 80120, USA; msontag@ciinternational.com (M.K.S.); jmiller@ciinternational.com (J.M.); ykellar-guenther@ciinternational.com (Y.K.-G.); 3 Colorado School of Public Health, University of Colorado, Anschutz Medical Campus. Aurora, CO 80045, USA; sarah.mckasson@cuanschutz.edu; 4 RTI International, Research Triangle Park, NC 27709, USA; sshone@rti.org

**Keywords:** newborn screening, consensus building, performance measures, quality improvement, quality indicators

## Abstract

Newborn screening is a public health program facilitated by state public health departments with the goal of improving the health of affected newborns throughout the country. Experts in the newborn screening community established a panel of eight quality indicators (QIs) to track quality practices within and across the United States newborn screening system. The indicators were developed following iterative refinement, consensus building, and evaluation. The Newborn Screening Technical assistance and Evaluation Program (NewSTEPs) implemented a national data repository in 2013 that captures the quality improvement metrics from each state. The QIs span the newborn screening process from collection of a dried blood spot through medical intervention for a screened condition. These data are collected and analyzed to support data-driven outcome assessments and tracking performance to improve the quality of the newborn screening system.

## 1. Introduction

Newborn screening (NBS) began in the United States in 1963 when four states began using blood drops collected on filter paper to detect Phenylketonuria (PKU) [1]. More than 55 years later, laboratory scientists still analyze dried blood spots using the same filter paper technology developed by Dr. Robert Guthrie in the 1960s. As of June 2019 all 50 states, Puerto Rico and the District of Columbia screen nearly 4 million babies per year for over 35 conditions included on a national Recommended Uniform Screening Panel (RUSP) [2].

The newborn screening test has moved beyond bloodspot testing to include hearing and congenital heart point of care testing. National policies over the past several decades have safeguarded funding while the establishment of federal programs at the Centers for Disease Control and Prevention (CDC) and the Health Resources and Services Administration (HRSA) have enhanced the system by establishing quality assurance, quality improvement, evidence review, advisory committee, and technical assistance related programs [1].

This simple, life-saving/quality of life enhancing test—the newborn screen—is backed by a complex system comprised of laboratory testing, follow-up, diagnosis, treatment or management, evaluation, and education [3]. Every state NBS program is made up of these six components: 1) Laboratory testing or screening, which includes the collection of dried blood spots (DBS) and screens for critical congenital heart disease and hearing loss at the birthing facility, transportation of the DBS to the NBS laboratory, testing the DBS at the NBS laboratory to identify infants at risk of one of the conditions on the state screening panel, and timely reporting out of the results to medical professionals. 2) Follow-up for the rapid location and referral of those at risk newborns by the state NBS program. 3) Diagnosis: medical evaluation and additional testing of screen-positive infants to make a definitive diagnosis or determine that the screening result was a false-positive. 4) Treatment or management by rapid planning and implementation of long-term therapy for infants diagnosed with a newborn screening disorder. 5) Evaluation: assessment of the previous four activities to identify opportunities for quality improvement and to gauge benefits to the patient, family, and society; and 6) Education of the parents, medical professionals, legislators, newborn screening personnel (lab and follow-up), and stakeholders. Tracking of outcomes and incorporating quality improvement measures is a critical component of program success. Public health laboratories in the United States analyze 97% of the country’s NBS tests [1]. The Association of Public Health Laboratories (APHL) is the national organization representing these state public health laboratories. APHL is also home to the Newborn Screening Technical assistance and Evaluation Program (NewSTEPs), a national technical assistance resource center funded by the Genetic Services Branch of the Maternal and Child Health Bureau at HRSA since 2012 to support NBS laboratory and follow-up systems to achieve data-driven quality improvements [4]. NewSTEPs provides access to a national data repository with peer-developed quality metrics and case definitions collected on a routine, scheduled basis. The NewSTEPs data repository is a secure database accessible to state newborn screening program staff with data ownership and reporting requirements guided by a data use agreement or memorandum of understanding [5]. The data repository offers a web-based forum for states NBS programs to enter quality indicator (QI) data, to securely compare their data against aggregate national and regional reports, to view trends in data over time, and to identify areas for improvement.

The recording, analyzing, and sharing of screening observations and measurements becomes necessary when evaluating the successes, gaps, and areas for data-driven improvements in a dynamic system that operates uniquely in each state, with factors such as fees and budgets, operating hours, screening methodologies, disorders screened, staffing workflows, and reporting algorithms varying from program to program. Acknowledging the unique qualities of each NBS program, in 2012, NewSTEPs took the lead in facilitating stakeholder engagement and consensus building to refine a set of uniform QIs, allowing programs to use standardized metrics to track performance, identify outliers, initiate data-driven improvements, perform gap analysis, and to engage in national and peer benchmarking. These NewSTEPs QIs are a reflection of the quality of the national NBS system where uniform measures were otherwise previously lacking.

## 2. Materials and Methods

The process of establishing, defining, and collecting uniform quality measures that would allow inter- and intra-program comparisons across multiple years required significant consensus-building and engagement from the newborn screening community, including representatives from the NBS laboratory, follow-up, and medical specialists. A meeting of subject matter experts to establish an initial set of QIs followed by pilot testing in one state’s newborn screening system yielded a framework from which the final eight QIs emerged. The final QIs- incorporate measurements from the full spectrum of the newborn screening system, from dried blood spot collection and specimen delivery to the laboratory, to point of care screening, diagnosis, and tracking missed (false negative) cases. Structured group communication methodologies, including the Delphi process, public comment, and World Café, were employed by NewSTEPs [6,7]. This step-wise and iterative community engagement process is outlined chronologically in Table 1.

### 2.1. Subject Matter Expert Meeting

In June 2011, APHL, in collaboration with the Genetic Alliance, convened 38 newborn screening experts representing a range of geographically diverse states at varying levels of screening complexity. In this instance, screening complexity referred to the number of disorders screened, the number of screens performed on each newborn (one versus two), and the number of annual births in each state (see Supplementary Materials for attendee list, states and organizations represented, and newborn screening program characteristics). The goal of this initial meeting was to develop a set of metrics, called QIs, which would be useful to newborn screening programs in evaluating their systems from a representative sample of NBS programs around the nation.

Historically, many states have collected and reported performance measures specific to the state processes, including specimen transit time from hospital to laboratory, the extent of missing demographic information on specimen collection cards, age of newborn at time of specimen collection, and others [8]. This exercise, however, was the first time a set of quantitative QIs were developed at a national level for standardization across all programs, allowing for state-to-state comparisons. Foundational data collection efforts of state newborn screening programs, including those provided to the National Newborn Screening and Genetics Resource Center (NNSGRC), proved useful in the establishment of the national newborn screening QIs [9].

The attendees of the June 2011 subject matter experts meeting addressed the following objectives: 1) identification and categorization of existing state-specific quality metrics; 2) harmonization of definitions of existing quality metrics; 3) summarization of the utility of state-specific performance measures and identification of new, standardized quality measures; 4) identification of common metrics collected by all participating states to be incorporated into the development of uniform national newborn screening QIs; and 5) definition of key QIs useful for measuring operational and outcome-based newborn screening processes.

In defining the ten QIs (Table 2) established during this meeting, the meeting facilitator used the World Café communication strategy [10] that fosters collaborative dialogue, sharing, and incorporation of multiple layers of thought and opinions through small-group engagement, followed by a small group voting mechanism to gauge the perceived utility and impact of the metrics.

### 2.2. Pilot Testing

In fall 2011, the New Jersey Newborn Screening Program and its divisional Quality Assurance Program participated in a pilot study to collect the 10 QIs (Table 2) developed during the first subject matter experts meeting to determine the feasibility and utility of the QIs. Data experts used a variety of built-in, user-defined result filters and queries to export key data elements to MS Excel, MS Access, and Crystal Reports for analyses by the New Jersey Department of Health Laboratory Information Management System, Neometrics (NatusMedical, Inc., San Carlos, CA, USA) [11].

### 2.3. Additional Subject Matter Expert Engagement

In July 2012, NewSTEPs convened the second meeting of 57 subject matter experts (see Supplementary Materials for attendee list) to review lessons learned from the New Jersey Newborn Screening Program pilot of the ten QIs and to obtain feedback on modifications required to adopt the most complete and useful set of performance metrics. NewSTEPs staff and meeting facilitators utilized an electronic polling system to perform a quantitative evaluation of attendees’ perceptions of each QI based on three criteria: 1) perceived importance, 2) appropriateness of the definition and 3) feasibility/challenges of collecting the data. The subject matter experts present during this meeting also reviewed the QIs together using a qualitative evaluation approach, where they utilized polling devices that recorded their answer choices on how important each one was, suggesting refinements and attaining consensus within the group.

In January 2013, NewSTEPs convened the third meeting of 11 subject matter experts (see Supplementary Materials for attendee list). This group finalized the twice refined-by-consensus QIs for public distribution, eventual data collection, and they consolidated the number of measures from 10 to 8.

### 2.4. Public Comment and Final Subject Matter Expert Engagement

In July 2013, NewSTEPs program staff distributed the final draft of QIs with their definitions as part of a survey (Table 3), and requested for comments using a public listserv that reached over 300 members of the newborn screening community, including: laboratorians, clinicians, follow-up staff, vendors, parents, advocacy groups, and other relevant stakeholders. The purpose of the NewSTEPs QI survey was to seek non-anonymized peer reviews and comments on each of the QIs proposed for national, standardized, annual data collection efforts. NewSTEPs issued a deadline of three weeks for stakeholders to complete the survey.

On December 5, 2015, a penultimate subject matter expert meeting convened electronically to refine and improve the conceptual definitions of the QIs and to ease the burden on states of entering quality indicator and case definition information into the NewSTEPs data repository. This workgroup of 31, was made up with a subset of attendees from the previous two meetings (July 2012 and January 2013), as well as previously unengaged stakeholders from the newborn screening community, including Laboratory Information Management Systems (LIMS) vendors (see Supplementary Materials for attendee list). During this meeting, NewSTEPs program staff and meeting facilitators employed the Delphi process to solicit feedback to reach agreement on the most recently revised QIs and the definitions that emerged following incorporation of public comments. The Delphi process is an iterative process used to generate consensus [13]. Typically, the Delphi starts with a solicitation of ideas from individuals working alone, most often in writing. The ideas gathered during the first iteration are then combined and presented to the larger group. The group then provides feedback and/or ratings until a consensus is reached. NewSTEPs staff distributed the first of two questionnaires electronically to each invited participant in advance of a scheduled in-person meeting, incorporating feedback into the QIs definitions within a source document where all changes and modifications were tracked.

NewSTEPs staff distributed the final draft of the QIs source document along with a second electronic questionnaire to invited participants with the expectation that the participants would provide their feedback during a final in-person meeting in February 2016 (see Supplementary Materials for attendee list). During this meeting, attendees discussed each QI, completed the second round of the Delphi process, and then arrived at consensus on the purpose and definition of each QI.

### 2.5. Ongoing Refinement of National Newborn Screening Quality Indicators

In January 2014, the Advisory Committee on Heritable Disorders in Newborns and Children’s (ACHDNC) tasked its Laboratory Standards and Procedures Workgroup with investigating the timeliness of newborn screening in the United States. The result was a series of recommendations later adopted by the Secretary of Health and Human Services, on the timeliness goals for newborn screening programs [12]. NewSTEPs incorporated these revised timeliness recommendations into Quality Indicator 5, as described in the Supplementary Materials (table titled “Revisions in 2014 and 2017 to National Newborn Screening Quality Indicator on Timeliness (Quality Indicator 5”)

In mid-2017, several newborn screening programs entering QI data into the NewSTEPs data repository notified NewSTEPs staff that a bias may exist when reporting timeliness measured in units of hours versus days. The bias occurs when reporting the following: 1) time elapsed from specimen collection to specimen receipt at the laboratory, 2) time elapsed from specimen receipt to reporting results, and 3) time elapsed from birth to reporting results. NewSTEPs staff assessed the severity and impact of this potential bias by analyzing data from three newborn screening programs that utilized different recording methods to acknowledge specimen receipt at the NBS laboratory. Results of the analyses confirmed that a bias does exist. Specifically, newborn screening programs that do not currently collect timestamps (e.g., time of receipt of specimen at laboratory) will enter data in integer values of whole units of days as opposed to units of hours for programs that do incorporate timestamps for laboratory receipt. To address this, NewSTEPs sought feedback from the subject matter experts who had been convened in February 2016 (Table 1) and ultimately modified QIs 5b, 5c, and 5d to units of days from hours. The full list of changes made to the Quality Indicator 5 elements between 2013 and 2017 can be found in the Supplementary Materials (table titled “Revisions in 2014 and 2017 to National Newborn Screening Quality Indicator on Timeliness (Quality Indicator 5”).

## 3. Results

### 3.1. Development of National Newborn Screening Quality Indicators

#### 3.1.1. *Subject Matter Expert Meeting*

Following the voting process, 16 proposed measures received more than five votes each, with some measures combined into subcategories of a larger measure (e.g., various metrics capturing timeliness of the newborn screening process). Based upon the outcome of each potential QI, its ability to track improvements in the newborn screening system, scientific merit, relevance to stakeholders, and feasibility of collection, a final list of ten QIs were selected by the meeting attendees as viable options for inclusion in a standardized, national set of performance measures (Table 2). The subject matter experts in attendance also concluded that implementation and tracking of national newborn screening QIs should occur on a voluntary basis as determined by each state and that additional consensus would be required around the standardization of terminology, units, and methodologies to count newborns for each measure. Meeting attendees also indicated that the newborn screening community would benefit from considering practical questions around a national data collection effort, including data ownership, oversight, transparency, and state support of standardized national newborn screening QIs.

#### 3.1.2. Pilot Testing

The New Jersey Newborn Screening (NBS) Program concluded that all 10 QIs proposed during the June 2011 subject matter expert meeting (Table 2) were relevant and useful. The New Jersey NBS Program indicated that routine monitoring of the proposed QIs and acting on results based on data trends could improve performance of the program. Results of the New Jersey Newborn Screening program pilot of the proposed QIs are outlined in Table 3.

As a result of the pilot study, the New Jersey NBS program also noted that it was likely that the feasibility of any given newborn screening program to provide data for each QI will vary on how the program information management systems are configured, how data are stored, and how data can be retrieved. The pilot study in New Jersey also revealed a need for additional education around each QI and its corresponding definition, as well as broader system based quality improvements that should be addressed, such as developing a better understanding of gaps in data around parent refusals for a newborn screen.

#### 3.1.3. Additional Subject Matter Expert Feedback

**Figure 1:**
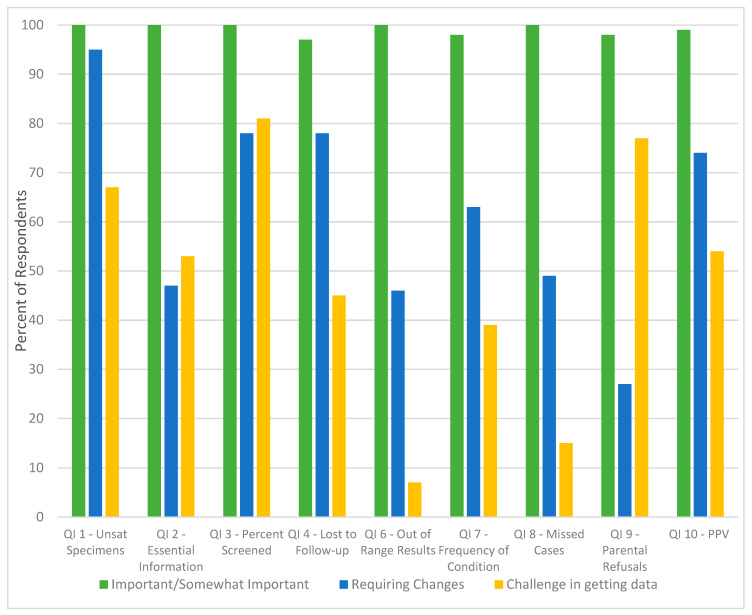
Electronic polling results from subject matter experts in response to importance, appropriateness, and challenges around 9 out of the ten proposed quality indicators (*n* = 57). Key for the QIs in Figure 1. Quality Indicator (QI) 1: Percent of unsatisfactory specimens due to improper collection. Quality Indicator (QI) 2: Percent of dried blood spot cards with all essential information. Quality Indicator (QI) 3: Percent of eligible infants receiving a valid newborn screen. Quality Indicator (QI) 4: Rate of loss to follow-up: unsatisfactory and out-of-range. Quality Indicator (QI) 6: Rate of out-of-range results, any referral to evaluation. Quality Indicator (QI) 7: Prevalence of condition detected at birth. Quality Indicator (QI) 8: Rate of missed cases (false negatives). Quality Indicator (QI) 9: Percent of parental refusals. Quality Indicator (QI) 10: Positive predictive value (PPV) of out-of-range results.

**Figure 2:**
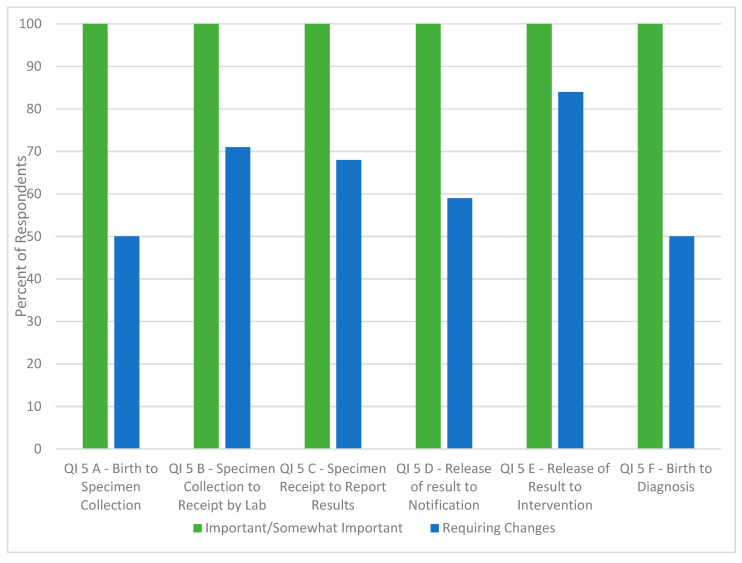
Electronic polling results from subject matter experts in response to importance of and degree of change required around proposed newborn screening timeliness specific quality indicators (*n* = 57). Key for the QIs in Figure 2. Quality Indicator (QI) 5a: Time elapsed from birth to specimen collection. Quality Indicator (QI) 5b: Time elapsed from specimen collection to receipt by laboratory. Quality Indicator (QI) 5c: Time elapsed from specimen receipt to reporting of results. Quality Indicator (QI) 5d: Time elapsed from release of results to notification of medical provider. Quality Indicator (QI) 5e: Time elapsed from release of results to medical intervention. Quality Indicator (QI) 5f: Time elapsed from birth to diagnosis.

As a result of the July 2012 subject matter expert meeting, the 10 proposed QIs benefited from additional refinement for clarity and the use of the Clinical Laboratory Standards Institute (CLSI) definitions [14], when appropriate, to ensure consistency in language around newborn screening system processes. Electronic polling results from the July 2012 meeting captured (Figure 1 and Figure 2) the perceived importance of each QI, appropriateness of the definition, and whether collecting the data may be a challenge. Each of the ten proposed QIs was deemed important or somewhat important by greater than 96% (*n* = 57) of all respondents. An average of 44% (*n* = 57, range = 0–81%)) of the respondents indicated that data on the majority of the proposed QIs would be challenging to collect. Further, the percentage of participants that found that the QI definition required changes ranged from 27–95% (Figure 1 and Figure 2).

The January 2013 subject matter expert meeting resulted in a consolidated list of QIs, reducing them from 10 to eight, with an accompanying purpose and definition articulated for each one. This was achieved by combining QI 3 (Percent of eligible infants receiving valid newborn screening test) and QI 9 (Percent of parental refusals), into a new QI 3 (Percent of eligible newborns not receiving a newborn screen, reported by dried blood spot or point-of-care screen(s)) while removing QI 10 (Positive predictive value (PPV) of out-of-range screening results) (Table 2 and Table 4).

In July of the same year, the NewSTEPs program sought public comment on the eight refined QIs from the newborn screening community (Table 5). NewSTEPs program staff reviewed, and incorporated as appropriate, comments received from a total of 52 individuals representing 32 US newborn screening programs, two international newborn screening programs, and one Laboratory Information Management Systems (LIMS) vendor.

The December 2015 and February 2016 subject matter expert meetings yielded more comprehensive and uniform definitions for the eight QIs, as well as a source document that provided a description, purpose, list of denominators, and definition for each quality indicator.

Table 6 highlights the results from round 1 and 2 of the Delphi process for QI 1, including percent agreement and changes that the meeting attendees proposed, and later accepted, as final for the National newborn screening QIs. Each QI (1 through 8) achieved a minimum of 81% agreement amongst the subject matter experts, after both rounds and all of the proposed changes to each QI were honored and incorporated by NewSTEPs.

### 3.2. Dissemination of National Newborn Screening Quality Indicators

In 2016, NewSTEPs adopted the final set of eight National Newborn Screening QIs (Table 4) following significant stakeholder input (Table 4).

The nuances associated with each QI, including numerator and denominator data, as well as sub-components were incorporated by NewSTEPs in a Quality Indicator source document. The source document also includes detailed definitions and a glossary of terms to eliminate any ambiguity regarding frequently used terminology [15]. QI quick tips are also available as a supplement to the QI Source Document on the NewSTEPs website to clarify common questions and definitions. All eight QIs are publically available to newborn screening programs within the NewSTEPs Website and Data Repository for annual data entry.

## 4. Discussion

To achieve consensus with national standardized QIs, the engagement of multiple stakeholders within the newborn screening community has been a necessary step. Numerous meetings for engagement coupled with utilizing a variety of communication and consensus-building techniques proved valuable in reviewing, refining, establishing, and operationalizing national performance measures for the newborn screening system. The lengthy process required six years of community engagement and resulted in very detailed and complex QIs. The complexity of these quality measures, however, make it necessary to ensure that ambiguity and misinterpretation of definitions are minimized, thereby assuring that data reported across all programs is comparable and is measuring the same processes across newborn screening systems.

### Collection of Quality Indicators

NewSTEPs’ mission is to achieve the highest quality newborn screening systems. The program will continue to review and monitor the national newborn screening QIs every five years to ensure continued feasibility and relevance. Quality indicator data is collected on an annual basis within the NewSTEPs data repository. State newborn screening programs are requested to provide data at least annually for each prior year, with submission accepted via either manual or automated data entry mechanisms. NewSTEPs has made data import templates available to newborn screening programs for ease of data entry as well as free data visualizations that NBS programs can utilize for quality improvement initiatives. The governance of all QI data submissions is overseen by a data use agreement or memorandum of understanding (MOU) between the Association of Public Health Laboratories and each state newborn screening program.

A recent demonstration of the utility of the standardized national newborn screening QIs is their use in a federal report developed by the US Government Accountability Office (GAO) on Newborn Screening Timeliness [16]. NewSTEPs collected QI 5 (timeliness) data from 40 newborn screening programs from 2012–2015 to deliver a comprehensive overview of newborn screening timeliness activities, as well as to fulfill a request for data from the GAO [17]. Utilization of the standardized QIs enabled NewSTEPs to collect and provide data with well-described consensus-based definitions, ensuring that data reported from one state would be comparable to data reported from any other state.

As newborn screening programs submit data, NewSTEPs welcomes feedback from NBS programs on the national newborn Screening QIs. NewSTEPs will use this feedback to ensure that the QIs continue to measure, in a harmonized fashion, the critical areas where quality improvements can be initiated within newborn screening systems. NewSTEPs plans to engage key stakeholders in an in-person meeting in the fall/winter 2019, in part to review feasibility of data collection and how this process can be made easier.

Despite significant efforts to elaborate all nuances and definitions in a Quality Indicator Source Document, there remain data elements that are open to interpretation or that may not be measured in a harmonized fashion across programs (i.e., capturing the receipt of a specimen as when the specimen was logged in the delivery area versus as when laboratory analysis was initiated on the specimen). These idiosyncrasies of programs will be realized and acknowledged as QI data and feedback is collected from all states and reviewed. As these areas for improvement are identified, they will be addressed and incorporated into modified QIs at five-year intervals.

Similar quality indicators or metrics are found in use by the Healthcare Improvement Scotland in a document titled “Pregnancy and newborn screening indicators” [18]. The document specifies a minimum set of high-level measures or indicators for pregnancy and newborn screening services in Scotland. These indicators are used for national consistency or local improvement within National Health Service boards in Scotland. The indicators specific to Newborn bloodspot screening (15–19) are very similar to the ones developed in the US in that they cover the areas of timeliness of services.

## 5. Conclusions

The efforts by newborn screening stakeholders between 2011 and present resulted in the first collection of a highly reviewed, consensus-based, refined set of national newborn screening QIs for use in newborn screening programs. These QIs can be used to evaluate and benchmark performance, make comparisons across programs that perform similar activities differently and allow for focused quality improvement initiatives. National newborn screening QIs are a necessary and useful tool in recording, analyzing and sharing observations and measurements within the newborn screening system, for tracking outcomes, and for implementing quality improvements.

## Figures and Tables

**Table 1:** Summary of Iterative Methods to Establish Newborn Screening Quality Indicators.

Date	Activity	Methodologies	Result
June 2011	Subject Matter Experts Meeting	Information Sharing World Café Affinity Diagrams Voting	10 QIs established
Fall 2012	Pilot Testing	Pilot testing of the 10 QIs in New Jersey	Understanding the feasibility of the collection and utility of the 10 QIs
July 2012	Subject Matter Experts Meeting	Agreement on definitions Qualitative consensus tracking Quantitative consensus tracking	Refinements to proposed QIs and associated definitions
January 2013	Subject Matter Experts Meeting	Consensus building	Reduction in number of QIs from 10 to 8; finalization of definitions of QIs
July 2013	Public Comment Period	Public comment requested on QIs, their utility and interpretation of definitions	Consensus building and community engagement
December 2015	Subject Matter Experts Meeting	Part one of a two-part Delphi process	Consensus on the purpose and definition of each QI
February 2016	Subject Matter Experts Meeting	Part two of Delphi process In-person feedback	Finalization of conceptual and operational definitions of the QIs

**Table 2:** 10 National Newborn Screening Quality Indicators, Developed June 2011 (unedited).

Data Element(s) Being Collected		Quality Indicator Definition
1	Percent of unsatisfactory specimens due to improper collection	Number of specimens on which laboratories cannot perform a complete newborn screening panel due to errors in collection divided by the number of specimens submitted
2	Percent of cards with all essential information	All dried blood spot (DBS) cards with state-defined essential information divided by all DBS cards received
3	Percent of eligible infants receiving a valid newborn screening test	Number of babies with a satisfactory and valid newborn screening result divided by number of live births in the same time period
4	Rate of loss to follow-up: unsatisfactory and out-of-range	Number of babies with an unsatisfactory specimen (and no previous or later satisfactory specimen) and out-of-range result that were lost to follow-up per state protocols at six months of age divided by the total number of infants in the state with unsatisfactory specimen and out-of-range test result.
5	Average time from: birth to specimen collection; specimen collection to receipt by laboratory; specimen receipt to reporting out results; release of out-of-range results to notification of medical provider; release of out-of-range results to medical intervention; birth to diagnosis	Number of specimens within each category divided by the summation of values in each time category.
6	Rate of out-of-range results, any referral to evaluation	Number of infants screened positive and need a repeat divided by the total number of infants screened; Number infants with the out-of-range result that need referral for evaluation divided by total number of infants screened
7	Prevalence of condition detected at birth: First screen versus Second screen	Number of infants confirmed affected based on an out-of-range first (versus subsequent) valid specimen divided by the number of infants screened
8	Rate of missed cases (false negatives)	Number of babies with disease who were not identified on newborn screening (but had a valid newborn screen) divided by all the babies who were screened and diagnosed with a disorder (true positives and false negatives combined).
9	Percent of parental refusals	Number of babies whose parents refused the complete newborn screening panel divided by the total number of live births
10	Positive predictive value (PPV) of out-of-range screening results	Number of babies with a “not normal” screen with a confirmed diagnosis divided by the number of all babies with a “not normal” screen; Number of babies referred for evaluation with a confirmed diagnosis divided by the number of all babies referred for evaluation

**Table 3:** New Jersey Newborn Screening Program quality indicator pilot study results.

Quality Indicator		New Jersey Newborn Screening Program Assessment of Feasibility and Utility
**1**	Percent of unsatisfactory specimens due to improper collection	The Newborn Screening Program was unable to differentiate between initial and repeat specimens (results include both). The program was also unable to exclude percent of specimens rejected per CLSI guidelines [12], specimens collected <24 h after birth or following a transfusion including those that were collected too soon due to state protocol. These specimens could not be delineated when providing data for Quality Indicator 1.
2	Percent of cards with all essential information	Results include all initial and repeat specimens that were initially received with missing essential demographic information even if the data was later provided. States that require fewer demographic fields are more likely to have better compliance with this QI.
3	Percent of eligible infants receiving a valid newborn screening test	Infants were identified by matching births in the state electronic birth certificate system (EBC) and the number of first specimens received by the laboratory, the number of unrepeated unsatisfactory first specimens, the number of “valid” first specimens (first specimen minus unrepeated). Results include initial specimens, repeat specimens with no previous specimen in the laboratory information management system (LIMS), and out of state births. In addition, actual patient data is not matched between the state EBC and LIMS.
4	Rate of loss to follow-up: unsatisfactory and out-of-range	This QI was feasible and straight forward to collect.
5	Average time from: birth to specimen collection; specimen collection to receipt by laboratory; specimen receipt to reporting out results; release of out-of-range results to notification of medical provider; release of out-of-range results to medical intervention; birth to diagnosis	Results for the time from the release of out-of-range results to notification of medical provider are presented as the time from receipt until a written report is issued by the laboratory. The immediate notification of critical results was not included; report date changes are made in the LIMS when duplicate reports are issued. The date of notification of medical provider was not recorded electronically, and thus results included both borderline cases (mailed letter) and presumptive cases (phone call). Results indicate that time to diagnosis varies significantly by disorder and stratification of results by disorder would be beneficial.
6	Rate of out-of-range results, any referral to evaluation	Results were beneficial as being described by the disorder to better describe assay performance and workload.
7	Prevalence of condition detected at birth: First screen versus Second screen	This QI was feasible and straight forward to collect.
8	Rate of missed cases (false negatives)	Results indicate that case definitions are important to determine false negatives.
9	Percent of parental refusals	The New Jersey Newborn Screening program was unable to calculate this QI at the time of the pilot study as this metric was being monitored by each birthing hospital and not the New Jersey Newborn Screening program.
10	Positive predictive value (PPV) of out-of-range screening results	Results indicate that calculations of PPV will vary based on a laboratory’s results categories and definition of out of range results and case definitions.

**Table 4:** National Newborn Screening Quality Indicators (Final as of 2016).

Quality Indicator Number	Data Element(s) Being Collected
1	Percent of dried blood spot specimens that were unacceptable due to improper collection and/or transport.
2	Percent of dried blood spot specimens with at least one missing state-defined essential data field upon receipt at the laboratory.
3	Percent of eligible newborns not receiving a newborn screen, reported by dried blood spot or point-of-care screen(s).
4	Percent of infants that have no recorded final resolution (confirmed diagnosis or diagnosis ruled out by an appropriate medical professional) with the newborn screening program.
5	Timeliness of newborn screening activities: a) Time from birth to specimen collection/ point-of-care testing. b) Time from specimen collection to receipt at your state’s newborn screening laboratory. c) Time from specimen receipt at your state’s newborn screening laboratory to reporting out specimen results. d) Time from birth to reporting out specimen results. e) Time from reporting out-of-range results to medical intervention by an appropriate medical professional for infants with a confirmed clinical diagnosis. f) Time from birth to confirmation of clinical diagnosis by an appropriate medical professional. g) For infants with an out-of-range newborn screen result requiring a clinical diagnostic workup by an appropriate medical professional, time from birth to time of determining if a result was a false positive.
6	Percent of infants with an out-of-range newborn screen result requiring clinical diagnostic workup reported by disorder category.
7	Percent of disorders detected by newborn screening with a confirmed diagnosis by an appropriate medical professional.
8	Percent of missed cases, reported by disorder.

**Table 5:** Questions asked in the NewSTEPs Quality Indicator Survey (July 2013).

Name:	
Organization Name:	
Email Address:	
Quality Indicator Specific Questions	For Each QI please respond to the following two questions:
Quality Indicator 1: Percent of invalid dried blood spot specimens/cards due to improper collection	Is this QI Important to capture? (Yes/No; if no, please specify why this Quality Indicator is not important) Is the definition for this QI concise and clear? (Yes/No; if no, please specify what changes would make this QI more relevant)
Quality Indicator 2: Percent of dried blood spot specimens/cards missing essential information	
Quality Indicator 3: Percent of eligible infants not receiving valid newborn screening test, reported by dried blood spot or point of care test(s)	
Quality Indicator 4: Percent of loss to follow-up	
Quality Indicator 5: Time elapsed from birth to screening, follow-up testing, confirmed diagnosis	
Quality Indicator 6: Percent of out of range results	
Quality Indicator 7: Prevalence of condition detected by newborn screening for each disorder	
Quality Indicator 8: Percent of missed cases (false negatives), reported by disorder	

**Table 6:** Summary of results from the Delphi process for Quality Indicator 1.

Question	Percent Agreement	Proposed Changes to the Quality Indicator
Quality Indicator 1: Percent of dried blood spot specimens that were unacceptable due to improper collection and/or transport.		
Question 1.1: Do you agree that the use of the term “unacceptable” is used correctly for this Quality Indicator?	100%	Add text in italics: requiring an additional sample *collected from the newborn* and submitted to the laboratory
Question 1.2: Should errors due to collection and errors due to transportation be reported separately as Quality Indicator 1a and Quality Indicator 1b?	78.6%	Improper transport definition has been amended to (in italics): *Any specimen received after the state-defined length of time that deems a specimen unacceptable for testing. In addition, the CLSI guidelines include placing specimens in a sealed plastic bag without a desiccant as a transport error.* Specimens that are not completely dry are now accounted for in errors due to collection.
Question 1.3: Should an additional quality indicator capture unacceptable specimens based on laboratory errors (e.g., DNA contamination)?	61.5%	None. Do not add new QI.
Question 1.4: Can you see opportunities for multiple interpretations using these definitions?	53.8%	Removed the word “card” in favor of using “specimen” throughout the document. Inclusion of the following phrase to the purpose statement: “and, therefore, requiring additional work for laboratory personnel to acquire an acceptable specimen.”

## Supplementary Materials

Supplementary materials can be found at https://www.mdpi.com/2409-515X/5/3/34/s1.
