# Supplementary material for: Development of National Newborn Screening Quality Indicators in the United States
Source: Int J Neonatal Screen. 2019 Sep 12;5(3):34. doi: 10.3390/ijns5030034 (PMC7510218; doi:10.3390/ijns5030034)
Supplement: Supplementary file 1 [file IJNS-05-00034-s001.pdf]

Supplementary Materials

Participants in the June 2011 Subject Matter Experts Meeting

Additional information includes state or organization represented by each participant, number of newborn screens performed in each represented state, number of annual births in each represented state, and number of core conditions screened in each representative state at the time of the meeting.

**Newborn Screening Subject Matter Experts Meeting on QIs June 14-15, 2011**				
**Attendee Name**	**State/Affiliation**	**Annual Births (2010) [1]**	**Number of Core RUSP Conditions Screened in 2011 [2]**	**One Screen or Two Screen State**
Cheryl Hermerath	Oregon	45,904	28	Two
Karim George	Kentucky	53,565	28	One
Scott Shone	New Jersey	103,932	28	One
Patrick Hopkins	Missouri	77,588	28	One
Mark McCann	Minnesota	68,269	28	One
Roger Eaton	Massachusetts	73,275	29	One
Michael Glass	Washington	86,507	28	Two
Michele Caggana	New York	246,081	29	One
Ward B. Jacox	Arizona	88,090	29	Two
Bob Bowman	Indiana	84,794	28	One
Bill Young	Michigan	113,509	29	One
Deborah Rodriguez	New York	246,081	29	One
Sharon Vaz	Oklahoma	52,347	27	One
Sheila Weiss	Washington	86,507	28	Two
Leslie Gafney	California	510,980	29	One
Christelle Larose	Rhode Island	11,843	28	One
Lou Bartoshesky	Delaware	11,682	28	Two
Judi Tuerck	Oregon	45,904	28	Two
Donna Williams	Texas	392,764	28	Two
Jane Getchell	Delaware	11,682	28	Two
National Partners				
Sharon Terry	Genetic Alliance			
Natasha Bonhomme	Genetic Alliance			
Michele Puryear	Health Resources and Services Administration (HRSA)			
Sara Copeland	HRSA			
Debi Sakar	HRSA			

Alaina Harris	HRSA	[redacted]
Carla Cuthbert	Centers for Disease Control and Prevention (CDC)	
Joanne Mei	CDC	
Marci Sontag	Colorado School of Public Health	
Alan Zukerman	National Institutes of Health (NIH)	
Rebecca Goodwin	NIH	
Swapna Abhyankar	NIH	
Reed Deshler	AlignOrg Solutions	
Mike Smith	AlignOrg Solutions	
Jelili Ojodu	Association of Public Health Laboratories (APHL)	
Elizabeth Jones	APHL	
Asha Farrah	APHL	

Participants in the July 2012 Subject Matter Experts Meeting

Additional information includes state or organization represented by each participant, number of newborn screens performed in each represented state, number of annual births in each represented state, and number of core conditions screened in each representative state at the time of the meeting

**Newborn Screening Subject Matter Experts Meeting on QIs July 30-31, 2012**				
**Attendee Name**	**State/Affiliation**	**Annual Births (2011) [3]**	**Number of Core RUSP Conditions Screened in 2012 [2]**	**One Screen or Two Screen State**
Swapna Abhyankar	National Library of Medicine (NLM)	[redacted]		
Cindy Ashley	Alabama	58,448	28	Two
Becky Bailey	North Dakota	10,106	28	One
Lou Bartoshesky	Delaware	11,023	29	Two
Linda Beischel	Montana	12,118	28	One
Stan Berberich	Iowa	38,702	28	One
Natasha Bonhomme	Genetic Alliance	[redacted]		
Bob Bowman	Indiana	83,227	29	One
Amy Brower	American College of Medical Genetics and Genomics (ACMG)	[redacted]		
Michele Caggana	New York	240,916	29	One
Colleen Clarke	Loisusana	62,642	28	One
Anne Comeau	Massachusetts	72,439	28	One
Sara Copeland	HRSA	[redacted]		
William Cramer	Pennsylvania	142,514	28	One

Hank Dorkin	Children's Hospital / Harvard Medical School			
Roger Eaton	Massachusetts	72,439	28	One
Lisa Feuchtbaum	California	503,755	30	One
Bryant Fortner	South Carolina	57,155	28	One
Lucy Fossen	South Dakota	12,104	28	One
Debra Freedenberg	Texas	382,727	29	Two
Jane Getchell	APHL			
Michael Glass	Washington	87,463	28	Two
Aaron Goldenberg	Case Western Reserve University			
Art Hagar	Georgia	130,280	28	One
Alaina Harris	HRSA			
Kathryn Hassel	University of Colorado, Denver			
Cindy Hinton	CDC			
Amy Hoffman	ACMG			
Philis Hoggatt	Mississippi	38,669	29	One
Patrick Hopkins	Missouri	75,446	28	One
Cindy Ingham	Vermont	6,009	28	One
**Ward Jacox**	**Arizona**	**86,441**	**28**	**Two**
Carol Johnson	Iowa	38,702	28	One
Yvonne Kellar-Guenther	Colorado School of Public Health			
Jamey Kendall	Kansas	40,341	28	One
Janice Kong	Hawaii	18,980	28	Two
Michelle Lewis	Johns Hopkins University			
Sharon Linard	Ohio	138,483	28	One
Jennifer Macdonald	Virginia	103,013	28	One
Mark McCann	Minnesota	68,772	28	One
Jelili Ojodu	APHL			
Susan Oliver	Connecticut	36,539	29	One
Richard Parad	Brigham and Women's Hospital			
Melissa Parisi	NIH			
Julie Raburn-Miller	Missouri	75,446	28	One
Deborah Rodriguez	New York	240,916	29	One
Inderneel Sahai	Massachusetts	72,439	28	One
Scott Shone	New Jersey	104,230	29	One
Marci Sontag	Colorado School of Public Health			
Susan Tanksley	Texas	382,727	29	Two
Laura Taylor	Colorado	65,187	29	Two
Lois Taylor	Florida	213,148	29	One
Patricia Terry	Mississippi	38,669	29	One
Tiina Urv	NIH			
Sheila Weiss	Washington	87,463	28	Two

Kupper Wintergerst	University of Louisville	[redacted]		
Alan Zuckerman	NLM	[redacted]		

Participants in the January 2013 Subject Matter Experts Meeting

Additional information includes state or organization represented by each participant, number of newborn screens performed in each represented state, number of annual births in each represented state, and number of core conditions screened in each representative state at the time of the meeting

**Newborn Screening Subject Matter Experts Meeting (Webinar) on QIs January 23, 2013**				
**Attendee Name**	**State/Affiliation**	**Annual Births (2011) [4]**	**Number of Core RUSP Conditions Screened in 2012 [2]**	**One Screen or Two Screen State**
Stan Berberich	Iowa	39,094	29	One
Michele Caggana	New York	236,980	29	One
Debra Freedenberg	Texas	387,340	29	Two
Ward Jacox	Arizona	85,600	28	Two
Alex Kemper	Duke University	[redacted]		
Jamey Kendall	Kansas	38,839	28	One
Sharon Linard	Ohio	138,936	29	One
Jennifer MacDonald	Virginia	102,147	28	One
Inderneel Sahai	Massachusetts	71,788	29	One
Lois Taylor	Florida	215,407	29	One
Tiina Urv	NIH	[redacted]		

Participants in the December 2015 and February 2016 Subject Matter Experts Meeting

Additional information includes state or organization represented by each participant, number of newborn screens performed in each represented state, number of annual births in each represented state, and number of core conditions screened in each representative state at the time of the meeting

**Newborn Screening Subject Matter Experts Meeting (Webinar) on QIs December 5, 2015 and February 10, 2016**				
**Attendee Name**	**State/Affiliation**	**Annual Births (2011) [5]**	**Number of Core RUSP Conditions Screened in 2012 [2]**	**One Screen or Two Screen State**
Willie Andrews	Virginia	103,303	31	One
Mei Baker	Wisconsin	67,041	31	One
Stan Berberich	Iowa	39,482	31	One
Lisa Canton	Oklahoma	53,122	31	One

Bob Currier	California	491,748	31	One
Sam (Sandra) Dawe	Wisconsin	67,041	31	One
Roger Eaton	Massachusetts	71,492	31	One
Amy Gaviglio	Minnesota	69,834	31	One
Fizza Gulamali-Majid	Maryland	73,616	30	Two
Kathy Hassell	Colorado	66,581	30	Two
Vincent High	5AM Solutions	[redacted]		
Patrick Hopkins	Missouri	75,061	31	One
Mike Johnston	PerkinElmer	[redacted]		
Mike Lemke	PerkinElmer			
Sarah McKasson	Colorado School of Public Health			
Joshua Miller	Colorado School of Public Health			
Jelili Ojodu	APHL			
Kostas Petritis	Arizona	85,351	30	Two
Ashleigh Ragsdale	Washington	88,990	31	Two
Walter Reichert	Natus	[redacted]		
Brendan Reilly	Texas	403,618	31	Two
Dari Shirazi	Iowa	39,482	31	One
Scott Shone	New Jersey	103,127	31	One
Sikha Singh	APHL	[redacted]		
Stuart Smiley	5AM Solutions			
Marci Sontag	Colorado School of Public Health			
Tricy Thomas	Natus			
Jodi Vaughn	PerkinElmer			
Johnna Watson	Maryland	73,616	30	Two
Alisha Wruck	Minnesota	69,834	31	One
Careema Yusuf	APHL	[redacted]		

Revisions in 2014 and 2017 to National Newborn Screening Quality Indicator on Timeliness (Quality Indicator 5)

Initial Quality Indicator 5 following inclusion of feedback from public comment period (2013)	Revised Quality Indicator 5 to adhere with ACHDNC timeliness recommendations (2014)	Revised Quality Indicator 5 to adhere to units of days versus hours modification requested by newborn screening programs (2017)
a) Birth to specimen collection, data collected in aggregate by state, with proportions of screens indicated in the following categories less	a) Time from birth to specimen collection/ point-of-care testing with the number of specimens/screens tallied in the following categories:<br>• Less than 12 hours from birth<br>• 12 to 24 hours from birth	**a) Time from birth to specimen collection/ point-of-care testing with the number of specimens/screens tallied in the following categories:**

than 12 hours, 12 to 24 hours, greater than 24 to 48 hours, greater than 48 to 72 hours, 4 days and greater<br>I. Initial dried blood spot screen:<br>II. Initial critical congenital heart disease screening<br>III. Initial early hearing loss screening<br>IV. For subsequent dried blood spot screen: less than 7 days, 7-10 days, greater than 10 to 14 days, greater than 14 days.<br><br>b) Specimen collection to receipt by lab [reflected by time sample is logged in at lab], data collected in aggregate by state, with proportions of screens indicated in the following categories: Same day as collection, calendar day after collection, 2 calendar days after collection, 3 calendar days after collection, 4 calendar days after collection, 5 - 6 calendar days after collection, more than 7-10 calendar days after collection.<br>I. Initial dried blood spot screen:<br>II. For subsequent dried blood spot screen	• Greater than 24 to 48 hours from birth<br>• Greater than 48 to 72 hours from birth<br>• Greater than 72 hours from birth<br>• Time elapsed unknown<br><br>i. Number of first dried blood spot specimens collected in the specified time intervals from birth, divided by the total number of first dried blood spot specimens collected. Total number of first dried blood spot specimens collected is calculated through the summation of values entered for each time interval category.<br>ii. Number of reported critical congenital heart disease (CCHD) screens completed in the specified time intervals from birth, divided by the total number of critical congenital heart disease (CCHD) screens. Total number of reported complete critical congenital heart disease (CCHD) screens is calculated through the summation of values entered for each time interval category.<br>iii. Number of reported early hearing detection and intervention (EHDI) screens completed in the specified time intervals from birth, divided by the total number of early hearing detection and intervention (EHDI) screens. Total number of reported complete early hearing detection and intervention (EHDI) screens is calculated through the summation of values entered for each time interval category.	Less than 12 hours from birth<br>12 to 24 hours from birth<br>Greater than 24 to 48 hours from birth<br>Greater than 48 to 72 hours from birth<br>Greater than 72 hours from birth<br>Time elapsed unknown<br><br>Total number of first dried blood spot specimens collected is calculated through the summation of values entered for each time category.<br><br>i. Number of first dried blood spot specimens collected in the specified time categories in units of hours from birth, divided by the total number of first dried blood spot specimens collected.<br><br>ii. Number of reported critical congenital heart disease (CCHD) screens completed in the specified time categories in units of hours from birth, divided by the total number of critical congenital heart disease (CCHD) screens.<br><br>iii. Number of reported early hearing detection and intervention (EHDI) screens completed in the specified time categories in units of hours from birth, divided by the total number of early hearing detection and intervention (EHDI) screens.

c) Specimen receipt to reporting out complete results, data collected in aggregate by state, with proportions of screens indicated in the following categories:<br>less than 12 hours, 12 to 24 hours, greater than 24 to 48 hours, greater than 48 to 72 hours, 4 days and greater.<br><br>d) Release of out-of-range results to notification of medical provider [reported by disorder], data collected in aggregate by state, with proportions of screens indicated in the following categories:<br>less than 12 hours, 12 to 24 hours, greater than 24 to 48 hours, greater than 48 to 72 hours, 4 days and greater.	iv. In two screen states, number of first dried blood spot specimens collected for the second screen in the following time intervals from birth, divided by the total number of first dried blood spot specimens collected for the second screen. Total number of first dried blood spot specimens collected for the second screen is calculated through the summation of values entered for each time interval category.<br>• Less than 7 days from birth<br>• 7 to10 days from birth<br>• 11 to 14 days from birth<br>• 15 days or more from birth<br>• Time elapsed unknown<br><br>v. Number of subsequent dried blood spot specimens collected in the specified time intervals from birth, divided by the total number of subsequent dried blood spot specimens collected. Total number of subsequent dried blood spot specimens collected is calculated through the summation of values entered for each time interval category.<br>• Less than 7 days from birth<br>• 7 to10 days from birth<br>• 11 to 14 days from birth<br>• 15 days or more from birth<br>• Time elapsed unknown<br><br>**b) Time from specimen collection to receipt by lab with the number of specimens tallied in the following categories:**<br>▪ Less than or equal to 24 hours after specimen collection<br>▪ Greater than 24 to 48 hours after specimen collection	iv. In two screen states, number of first dried blood spot specimens collected for the second screen in the following time categories in units of days from birth, divided by the total number of first dried blood spot specimens collected for the second screen.<br><br>Less than 7 days from birth<br>7 to10 days from birth<br>11 to 14 days from birth<br>15 days or more from birth<br>Time elapsed unknown<br><br>v. Number of subsequent dried blood spot specimens collected in the specified time categories in units of days from birth, divided by the total number of subsequent dried blood spot specimens collected.<br>Less than 7 days from birth<br>7 to10 days from birth<br>11 to 14 days from birth<br>15 days or more from birth<br>Time elapsed unknown<br><br>**b) Time from specimen collection to receipt by lab with the number of specimens tallied in the following categories:**<br>• Same day as collection (Day 0)<br>• Day after collection (Day 1)<br>• Day 2 after collection (Day 2)<br>• Day 3 after collection (Day 3)<br>• Day 4 after collection (Day 4)<br>• Day 5 after collection (Day 5)<br>• Day 6 after collection (Day 6)<br>• Greater than or equal to Day 7 after collection (>=Day 7)<br>• Time elapsed unknown

	▪ Greater than 48 to 72 hours after specimen collection<br>▪ Greater than 72 to 96 hours (4 days) after specimen collection<br>▪ Greater than 96 (4 days) to 120 hours (5 days) after specimen collection<br>▪ Greater than 120 (5 days) to 144 hours (6 days) after specimen collection<br>▪ Greater than 144 hours (6 days) after specimen collection<br>▪ Time elapsed unknown<br>i. Number of first dried blood spot specimens received at your state's newborn screening laboratory in the specified time intervals <u>from specimen collection</u>, divided by the total number of first dried blood spot specimens received at your state's newborn screening laboratory. Total number of first dried blood spot specimens received is calculated through the summation of values entered for each time interval category.<br>ii. Number of subsequent dried blood spot specimens received at your state's newborn screening laboratory in the specified time intervals <u>from specimen collection</u>, divided by the total number of subsequent dried blood spot specimens received at your state's newborn screening laboratory. Total number of subsequent dried blood spot specimens received is calculated through the summation of values entered for each time interval category.	Total number of first dried blood spot specimens received is calculated through the summation of values entered for each time category.<br><br>i. Number of first dried blood spot specimens received at your state's newborn screening laboratory in the specified time categories in units of days from specimen collection, divided by the total number of first dried blood spot specimens received at your state's newborn screening laboratory.<br>ii. Number of subsequent dried blood spot specimens received at your state's newborn screening laboratory in the specified time categories in units of days from specimen collection, divided by the total number of subsequent dried blood spot specimens received at your state's newborn screening laboratory.<br><br>**c) Time from specimen receipt at your state's newborn screening laboratory to reporting out specimen results, with the number of specimens tallied in the following categories (includes all first and subsequent specimens):**<br>Same day as receipt at lab (Day 0)<br>Day after receipt at lab (Day 1)<br>Day 2 after receipt at lab (Day 2)<br>Day 3 after receipt at lab (Day 3)<br>Day 4 after receipt at lab (Day 4)<br>Day 5 after receipt at lab (Day 5)

	**C) Time from specimen receipt at your state's newborn screening laboratory to reporting out specimen results, with the number of specimens tallied in the following categories (includes all first and subsequent specimens):**<br>▪ Less than 12 hours after receipt<br>▪ 12 to 24 hours after receipt<br>▪ Greater than 24 to 48 hours after receipt<br>▪ Greater than 48 to 72 hours after receipt<br>▪ Greater than 72 to 96 hours (4 days) after receipt<br>▪ Greater than 96 (4 days) to 120 hours (5 days) after receipt<br>▪ Greater than 120 (5 days) to 144 hours (6 days) after receipt<br>▪ Greater than 144 hours (6 days) after receipt<br>▪ Time elapsed unknown<br><br>i) For time-critical disorders<br>ii) For non-time-critical disorders<br>iii) Normal and out-of-range results for all disorders **from first dried blood spot specimens**<br>iv) Normal and out-of-range results for all disorders **from subsequent dried blood spot specimens**<br>v) In two screen states, normal and out-of-range results for all disorders **from second screen dried blood spot specimens**<br>**d) Time from birth to reporting out specimen results, with the**	Day 6 after receipt at lab (Day 6)<br>Greater than or equal to Day 7 after receipt at lab (>=Day 7)<br>Time elapsed unknown<br><br>Total number of first dried blood spot specimens received is calculated through the summation of values entered for each time category.<br><br>i) For time-critical disorders<br>ii) For non-time-critical disorders<br>iii) For normal and out-of-range results for all disorders from first dried blood spot specimens<br>iv) For normal and out-of-range results for all disorders from subsequent dried blood spot specimens<br>v) In two screen states, normal and out-of-range results for all disorders from second screen dried blood spot specimens<br><br>**d) Time from birth to reporting out specimen results, with the number of specimens tallied in the following categories (includes all first and subsequent specimens):**<br>Less than or equal to Day 2 after birth (<=Day 2)<br>Day 3 after birth (Day 3)<br>Day 4 after birth (Day 4)<br>Day 5 after birth (Day 5)<br>Day 6 after birth (Day 6)<br>Day 7 after birth (Day 7)<br>Day 8 after birth (Day 8)<br>Day 9 after birth (Day 9)<br>Greater than or equal to Day 10 after birth (>=Day 10)<br>Time elapsed unknown

	**number of specimens tallied in the following categories (includes all first and subsequent specimens):**<br>a. Less than or equal to 48 hours after birth<br>b. Greater than 48 to 72 hours after birth<br>c. Greater than 72 to 96 hours (4 days) after birth<br>d. Greater than 96 hours (4 days) to 120 hours (5 days) after birth<br>e. Greater than 120 hours (5 days) to 144 hours (6 days) after birth<br>f. Greater than 144 (6 days) to 168 hours (7 days) after birth<br>g. Greater than 168 hours (7 days) to 192 hours (8 days) after birth<br>h. Greater than 192 hours (8 days) to 216 hours (9 days) after birth<br>i. Greater than 216 hours (9 days) to 240 hours (10 days) after birth<br>j. Greater than 240 hours (10 days) after birth<br>k. Time elapsed unknown<br>i) For time-critical disorders<br>ii) For non-time-critical disorders<br>iii) Normal and out-of-range results for all disorders **from first dried blood spot specimens**<br>iv) Normal and out-of-range results for all disorders **from subsequent dried blood spot specimens**<br>In two screen states, normal and out-of-range results for all disorders **from second screen dried blood spot specimens**	Total number of dried blood spot specimens with out-of-range results requiring clinical diagnostic workup by an appropriate medical professional for time critical disorders is calculated through the summation of values entered for each time category.<br>i) For time-critical disorders<br>ii) For non-time-critical disorders<br>iii) For normal and out-of-range results for all disorders from first dried blood spot specimens<br>iv) For normal and out-of-range results for all disorders from subsequent dried blood spot specimens<br>v) In two screen states, normal and out-of-range results for all disorders from second screen dried blood spot specimens

REFERENCES

1- Centers for Disease Control and Prevention. National Center for Health Statistics (NCHS). Retrieved June 25, 2019 from https://www.cdc.gov/nchs/

2- Health Resources and Services Administration. Recommended Uniform Screening Panel (RUSP). Retrieved June 25, 2019 from https://www.hrsa.gov/advisory-committees/heritable-disorders/rusp/index.html

3- Martin JA, Hamilton BE, Osterman MJK, et al. Births: Final data for 2012. National vital statistics reports; vol 62 no 9. Hyattsville, MD: National Center for Health Statistics. 2013. Retrieved June 25, 2019 from https://www.cdc.gov/nchs/data/nvsr/nvsr62/nvsr62_09.pdf

4- Martin JA, Hamilton BE, Osterman MJK, et al. Births: Final data for 2013. National vital statistics reports; vol 64 no 1. Hyattsville, MD: National Center for Health Statistics. 2015. Retrieved June 25, 2019 from https://www.cdc.gov/nchs/data/nvsr/nvsr64/nvsr64_01.pdf

5- Martin JA, Hamilton BE, Osterman MJK, et al. Births: Final data for 2015. National vital statistics report; vol 66, no 1. Hyattsville, MD: National Center for Health Statistics. 2017. Retrieved June 25, 2019 from https://www.cdc.gov/nchs/data/nvsr/nvsr66/nvsr66_01.pdf
